# Value and Vulnerability Assessment of a Historic Tomb for Conservation

**DOI:** 10.1155/2014/357679

**Published:** 2014-07-07

**Authors:** Aykut Erkal, Hakki O. Ozhan

**Affiliations:** Department of Civil Engineering, Istanbul Kemerburgaz University, Mahmutbey Dilmenler Caddesi, No. 26, Bagcilar, 34217 Istanbul, Turkey

## Abstract

Monumental tombs reflect various social, cultural, architectural, religious, economic, and engineering features of a community. However, environmental weathering, natural disasters, poor maintenance, vandalism, and misuse unfortunately pose serious threats to these cultural assets. Historic monuments are often exposed to the highest risk due to their vulnerability. The Ottoman-style Nişancı Hamza Paşa tomb located in Karacaahmet Cemetery, Istanbul, the largest and oldest public cemetery in Turkey, is a case in point. The tomb consisting of six granite columns and a brick dome supported by six arches was constructed in 1605. Cracks, material loss, and decay as a result of adverse environmental effects and past earthquakes are evident. Therefore, this paper analyses the overall value of the tomb with respect to its historical, communal, evidential, and aesthetic aspects. Using the finite element approach and data on the tomb's material properties, a structural analysis under the self-weight and a time history analysis based on the earthquake ground motion data recorded in Duzce, Turkey, in November 1999 were conducted to encourage the conservation of this tomb and similar cultural heritage assets all over the world. The damage observed in the structure is congruent with the analysis results.

## 1. Introduction: Conservation of Historic Tombs in Cemeteries

Cultural heritage has been severely threatened by the increase in the magnitude and frequency of natural disasters [1] and deteriorating environmental effects over time [2]. The significance of cultural assets especially increases in areas where the richness of cultural heritage has the ability to motivate information production and cultural tourism [3]. Cemeteries are regarded as one of the vital components of the cultural heritage of a society since they accommodate special and significant landmarks in its history. They also convey information about social elements of a community. Being commemorative places of the dead, great respect is paid by the families and friends of the buried people. Moreover, the general public visit cemeteries to find peace and to study the history. In this regard, a cemetery can be regarded as a record of social history of an area [4]. Inscriptions on monuments can reveal significant truths about the past. Even its design and maintenance can convey a sense of the evolution of a society over time. These factors make cemeteries a valuable resource for research purposes. Due to their spiritual, commemorative, and social nature, the task of designing and maintaining them has always been one of importance. In the design, natural features are utilized to form aesthetic landscapes consisting of avenues, footpaths, boundaries, burial ground, gateways, trees, shrubs, herbaceous plants, and, most importantly, special architectural structures such as chapels, shelters, lodges, walls, and monuments [5]. Tombs, being commemorative monuments, are constructed to house the remains of notables of the community. Therefore, each tomb has a unique and special history and story. Almost in every cemetery, monumental tombs of various sizes exist, each of which has some unique magnitude of significance. As such, the inscriptions on tombs, their architectural design and engineering features, and the surrounding landscape cast light on many past events and traditions of a society [4]. Therefore their careful maintenance and conservation are essential. Conservation can be defined as the arrangements planned to minimize decay and damage, stabilize artefacts, and prevent or retard further deterioration [6].

**Figure 1:**
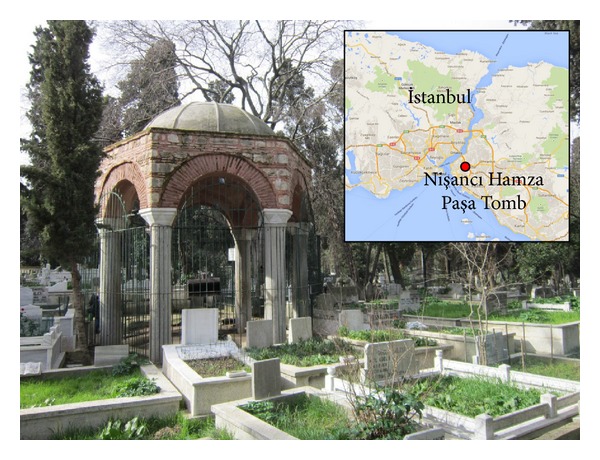
Nişancı Hamza Paşa tomb in Karacaahmet Cemetery.

Karacaahmet Cemetery, the oldest in Istanbul, was built almost 700 years ago in Üsküdar, a borough on the Asian side of Istanbul (Figure 1). Having an area of 3 km^2^, the cemetery is also the largest burial ground in Turkey. Its name, Karacaahmet, comes from the name of the warrior companion of Orhan I, second sultan of the Ottoman Empire. It is estimated that over a million people are buried in the cemetery [7] that contains many tombs constructed during the Ottoman Empire period. One of the monumental tombs located in this cemetery is the Nişancı Hamza Paşa tomb shown in Figure 1.

## 2. The Aim and the Methodology of the Study

Being an important cultural heritage structure, Nişancı Hamza Paşa tomb has historical, evidential, communal, and aesthetic values which constitute its significance. However, its longevity has caused the tomb to experience various strong ground motions and deteriorating atmospheric effects. Cracks and decay as a result of these adverse impacts on the structure are rather evident. This might possibly pose a threat to the future state of the tomb. In order to assess the seismic risk of a monumental structure, apart from hazard, evaluation of exposure and vulnerability of the structure is also required. That is, while hazard is related to the possibility of future seismic action and the seismic activity of a region can be conveniently described by means of seismic hazard maps, exposure and vulnerability need to be evaluated based on the structure since vulnerability is related to the structure's weakness and exposure is related to its value [8]. Therefore the aim of this study is to present a value analysis with respect to historical, evidential, communal, and aesthetic values of the tomb and to make a vulnerability appraisal utilizing finite element method (FEM) analysis tools to assess the potential risk. Although comprehensive value analysis is missing in many risk evaluations, it is indispensable since it is not only the historic structure itself that should be conserved but also the cultural, social, religious, or artistic significance that it has [4].

## 3. Value Analysis of Nişancı Hamza Paşa Tomb

The significance of a historic structure comprises all the diverse cultural and natural heritage values which prompt people to respond to it. These values tend to grow in strength and complexity over time, as understanding deepens and people's perceptions of a structure evolve. A framework for the assessment of the significance of all historic assets can be set based on four component values [9].

### 3.1. Historical and Evidential Value

Historical value comes from the elements that connect past people, events, and aspects of life to the present through a place. It refers to the illustrative or associative values of an asset. In practice, however, much of the historical value of an asset is not separable from its evidential value that is based on the evidence of past activities [10]. The tomb does not have an inscription and thus it is difficult to determine its architect and the exact construction time. However, Istanbul cultural heritage and cultural economics inventories indicate that the construction year of the tomb is 1605 [11]. The tomb was built for Nişancı Hamza Paşa, an Ottoman marksman living in the 16th century who was interred in the grave in the tomb on his death in 1605 [12]. Interestingly, this tomb is also known to the public as the “horse grave” as it is believed that the tomb was built for the horse of Sultan Karacaahmet who had a deep affection for his horse with whom he fought in most battles. However, Haskan does not confirm this [12].

### 3.2. Communal Value

Communal value derives from the meanings of a place for the people who relate to it or for whom it figures in their collective experience or memory [9]. Many people regard the tomb as a holy place and often visit and pray to God in the hope that their wish will be granted. It is believed that praying for a wish at a religious and spiritual place such as at a tomb of a mystic or an ancestor is a good deed. Thus, the tomb has a considerable communal value that is additionally strengthened by the people who believe that the horse of Karacaahmet Sultan will bring them luck if they visit the place and pray.

### 3.3. Aesthetic Value

**Figure 2:**
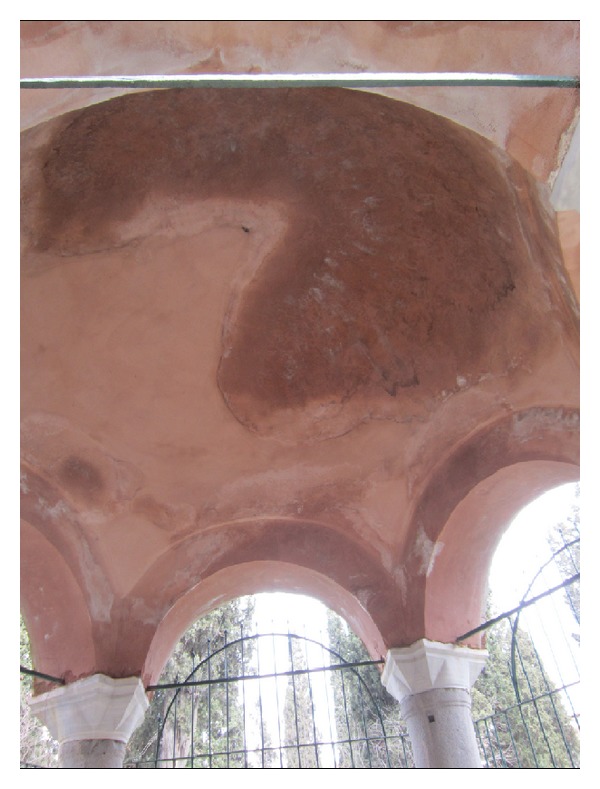
The interior of the dome.

The aesthetic value of the tomb is evident. Its plain architectural beauty greets the visitors as soon as they enter the cemetery. As an example of the classical Ottoman style, the tomb has a hemispherical dome (Figures 1 and 2). In most of the Ottoman style monumental structures, the dome, being a significant structural and aesthetic element, dominates the interior as much as it dominates the exterior. The interior of the dome symbolizes the spiritual world by providing space and thus a feeling of ascension to God [13]. The inner high ceiling of the tomb lends a spacious air to the interior giving a sense of the inaccessibility of God (Figure 2). The tambour of the tomb is composed of brick and stone masonry. The brick arches supported by six granite columns harmonize very well and lend a noble air. The plain but glorious look of the tomb accounts for its aesthetic value.

All the values attached to the tomb constituting its significance led to an evaluation of the effects of gravitational and earthquake loads on the structure to investigate the vulnerability of the monumental structure. Thus the building is simulated using the finite element method (FEM).

## 4. Finite Element Modelling

The dome is the significant structural and aesthetic element of the tomb. Such structures with domes have been analysed by various researchers. The comparison of the results between the shell theory and the finite element models for dome structures proved that finite element results are reliable. However, they made certain points worthy of attention in terms of uniform and symmetrical meshing, precise application of constraints, and compatibility of element boundaries to obtain well distributed force, moment, and stress resultants [14, 15]. Experimental studies were proved to be in agreement with the theoretical analysis as well [16]. Even dynamic behaviour of a hemispherical reticulated dome against rigid and deformable impacts was analysed by using a finite element approach. The damage modes such as global collapse and shear failure of the components of the dome were investigated by three-dimensional finite element method (FEM) simulations [17]. Being similar structures to some of the tombs, minarets, cylindrical tower-like slender structures, were analysed as well. The west minaret of Dolmabahce Mosque in Istanbul, Turkey, was analysed to ensure seismic safety and to evaluate the proposed strengthening method. The application of fibre-reinforced wrap around the critical cross-sections was suggested to improve the lateral behaviour and thus to provide seismic protection. Similar to the study herein, seismic loads were applied using linear elastic time history analysis for two different artificial ground motions and modal analysis was performed on the stone masonry [18].

**Figure 3:**
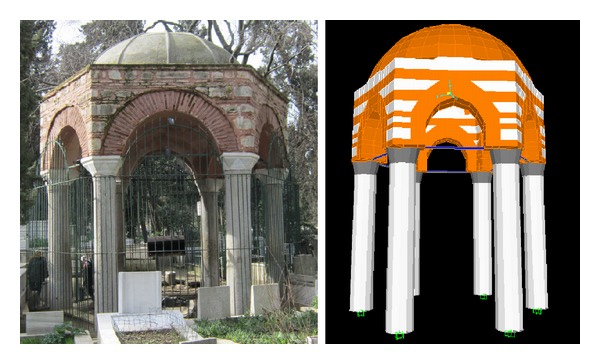
Nişancı Hamza Paşa tomb and its FEM simulation.

In order to see the dynamic behaviour of the masonry structure, a finite element model of Nişancı Hamza Paşa Tomb is generated (Figure 3). Modal analysis considering the first 24 modes, structural analysis under gravitational loads, and linear time history analysis based on the earthquake ground motion record taken at Duzce have been performed as a commonly used analysis approach [19]. Characteristics of the earthquake record used in the analysis are given in Table 1. Time step of the record is 0.005 sec and the first 6000 data points were taken into account in the analysis. The tomb structure is modelled in SAP2000-V10 [20]. During the linear time history analyses, the linear elastic force-deformation relationships were used. It is apparent that, at the time of the construction, only gravitational loads were taken into account. The large cross-sections of the tomb, in general, imply its overdesign. Therefore the finite element model assumes linear elastic material behaviour and ignores stiffness degradation.

The hemispherical dome is made of brickwork and carried by a hexagonal tambour, composed of limestone blocks and brick work placed in layers. This technique not only provides an optimum fit of masonry blocks but also embellishes the outer appearance of the tomb. Each side of the masonry tambour contains an arch made of brickwork to safely transfer the loads to the ground through granite columns. To facilitate load transfer from the arches to the columns, marble caps were used between the columns and arches (Figure 3).

The tomb is 6.70 m high (from the ground to the apex of the dome) with 6 columns, surrounding a circle with a radius of 1.9 m and placed at a distance of 2.2 m from each other. Each column has a height of 3.20 m and a radius of 0.27 m. The dome and the tambour were modelled using three-dimensional, 8-node, solid elements, while the caps, the columns, and the steel struts to laterally strengthen the columns are simulated as frame elements. Prismatic and nonprismatic frame elements have been used to construct the columns and the caps at the top of the columns, respectively. In the analyses, only horizontal ground motion was considered. In the FEM model of this monumental tomb, 2643 points, 18 frames, and 1608 solid elements have been generated. A picture of the tomb and its finite element model appears in Figure 3.

The dynamic equilibrium equation of the model structure can be stated as

(1) $$\begin{array}{c} M\ddot{u}+C\dot{u}+Ku=-M{\ddot{v}}_{g}, \end{array}$$

where *M*, *C*, and *K* are mass, viscous damping, and stiffness matrices of the structure; *u* is the displacement vector of the structure; and ${\ddot{v}}_{g}$ is the earthquake ground motion. A viscous damping of five percent was assumed for all vibration modes.

## 5. Determination of the Mechanical Properties of Tomb Materials

**Figure 4:**
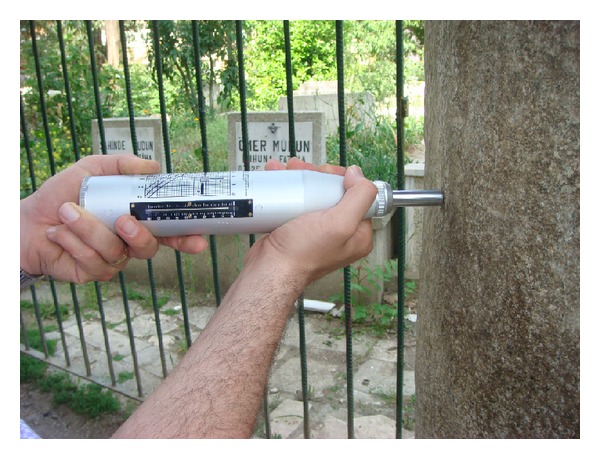
Schmidt hammer testing.

One of the major challenges of examining historic structures is the mechanical characterisation of materials for assessment and numerical modelling. Strength and elastic properties of the tomb's materials, herein, were determined considering the similar structures and based on Schmidt hammer testing due to its rapid, easy, and, most importantly, nondestructive nature (Figure 4). Testing was conducted closely in accordance with EN 12504-2:2012 [21]. During the application of the technique, certain precautions, however, need to be taken; for example, tested stone should be apparently elastic and should not disintegrate under the impact of the hammer and measurements should be carried out on smooth surfaces [22]. Paying attention to these issues, researchers have reported satisfactory correlations between Schmidt hammer rebound number and physical properties of stones [23]. In this study, a device with impact energy of 2.207 Nm was used. Such devices are commonly applied for the determination of stone properties [24]. Based on the hardness of the stone surface, granite, marble, and limestone, properties were determined with acceptable reliability. Empirical correlations suggested by Katz et al. [22] were employed to estimate Young's modulus, uniaxial compression strength, and unit weight ((2), (3), and (4)). The range of tensile strength values of the materials was also estimated considering the study by Vasconcelos et al. [25] (5). In accordance with Hooke's Law, shear modulus values were calculated. It should be noted that the aim was to estimate general in situ material properties by the testing despite the heterogeneity of the materials:

(2) $$\begin{array}{c} \ln\left(E\right)=-8.967+3.091\times \ln\left(N\right)\;\left(\text{in}\;\text{GPa}\right), \end{array}$$

(3) $$\begin{array}{c} \gamma =-2874+1308\times \ln\left(N\right)\;\left(\text{in}\;\text{kg}/{\text{m}}^{3}\right), \end{array}$$

(4) $$\begin{array}{c} \ln\left(f_{c}\right)=0.792+0.067\times N\;\left(\text{in}\;\text{MPa}\right), \end{array}$$

(5) $$\begin{array}{c} f_{t}=0.00106\times e^{0.118\times N}\;\left(\text{in}\;\text{MPa}\right), \end{array}$$

where *E* is Young's modulus, *γ* is the unit weight, *f*_*c*_ is compression strength, *f*_*t*_ is tensile strength, and *N* is the Schmidt hammer rebound number.  Table 2 summarises the obtained material properties.

## 6. Results and Discussion

Researchers adopt different approaches to the numerical modelling of masonry structures with regard to the objectives of their studies. Detailed micromodelling is generally used to provide a close representation of the local behaviour of masonry structures based on the detailed experimental data since the approach uses continuum elements for units and mortar, and discontinuum elements for their interface [26]. For the sake of simplicity, discontinuum elements are used to account for the behaviour of mortar joints only, in the simplified micromodelling approach. Employing average masonry properties, however, the structure is modelled with a single material in the macromodelling approach [27]. This approach is used widely in the global analyses of large-scale masonry structures since it requires considerably less time and less computational expense [28]. Therefore, macromodelling is facilitated in this study during the holistic numerical analysis of the entire tomb, assigning the properties of brick masonry, stone masonry, and single material elements to the relevant parts of the structure. The main challenge in the numerical modelling of masonry structures is the variability of its physical properties even in a specific structure due to the heterogeneity of the materials [29]. This is especially true for the historic masonry structures since the environmental impact could substantially change the mechanical properties of the structural materials. The common feature of the mechanical behaviour of masonry structures is their low tensile strength which can cause cracks. The unknown location, extent, and orientation of the existing cracks are the other reasons to use a macromodelling approach considering the unity of the current tomb structure.

The results of structural analysis under gravitational loads, modal analysis, and linear time history analysis have been presented and discussed in the following sections.

### 6.1. Self-Weight

**Figure 5:**
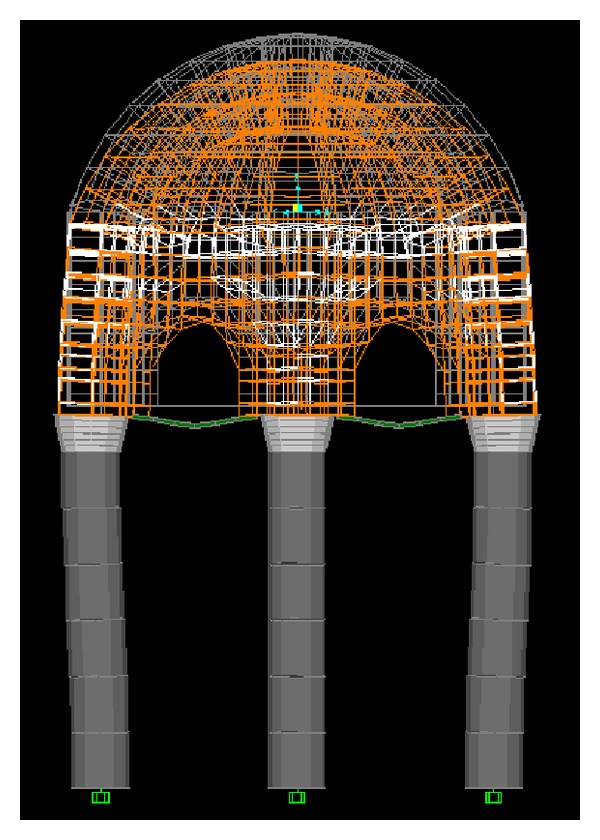
Deformation pattern under static gravitational loads.

The overall deformation behaviour of the tomb due to gravitational loads appears to be downward sag (Figure 5). The maximum vertical displacement of 0.07 mm at the centre of its dome can be attributed to the high vertical stiffness of the structure. Large dimensions of the tomb provide high stiffness. On the other hand, they cause large internal stresses due to the resulting large gravitational loads.

**Figure 6:**
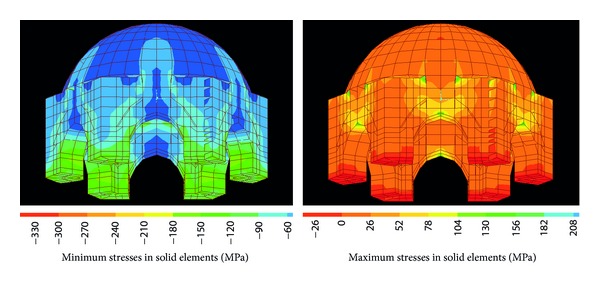
Stress contours in the dome of the tomb.

Figure 6 illustrates the minimum and maximum principal stresses developed in the dome and the tambour under the action of gravitational loads. As expected, minimum principal stresses especially increase in the tambour as the walls around the tambour carry the dome and reach the maximum value at locations where they meet columns. Minimum principal stress values of even up to 150–180 MPa develop in a very small region at the inner face of the tambour due to its bending behaviour.

Dome systems were extensively used in historic structures since domes efficiently cover the maximum space without any columns in the middle. The domes use compressive forces to transfer the gravity loads. Loads are usually distributed to the foundation with almost no bending in the shell, in a three-dimensional direction. Therefore very low tensile stresses develop thanks to its circular geometry. Because of this, the principal maximum stresses in the dome of the tomb seem considerably low. However, due to the bending of the arches around the dome, the maximum principal stresses at the bottom of the arches are in a very narrow range of between 70 and 100 Mpa. Although these stresses seem large, they can be accommodated through fine cracks. Similar stress patterns were also observed by Perucchio and Brune during the structural analysis of a cross-vaulted structure, the Great Hall in Rome, under static gravitational loads [30]. Stresses at the bottom of the vaults of the Great Hall exceed the tensile strength of material while the building stands stably. In general, all compressive stresses in this study are well within the compressive strength of marble and granite in the columns. Therefore, the structural system of the tomb does not statically pose a threat to the stability of the monument under the gravitational loads only. To investigate the lateral resistance of the monument, which is comprised of slender columns on which a relatively massive dome resides, dynamic analysis was performed.

### 6.2. Modal Analysis

**Figure 7:**
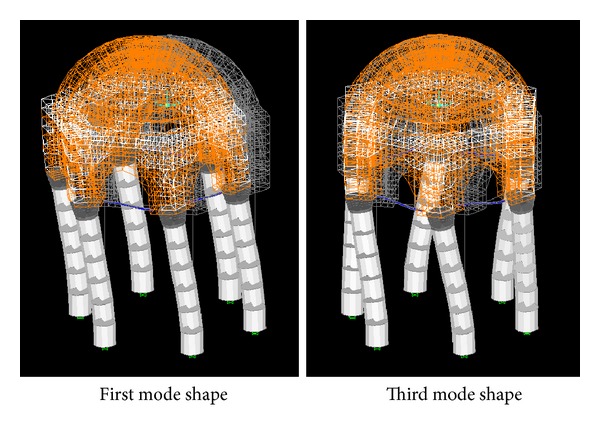
First and third mode shapes of the structure.

Any structure can vibrate in various ways. Each way of vibration is a different mode of the structure, having its own frequency. Modal analysis of the tomb has been conducted taking the first 24 modes into account. Total dynamic modal participation ratios of 99.7% in the *x*- and *y*-direction (perpendicular horizontal directions) and 99.9% around the *z*-axis (vertical direction) are obtained. The periods and frequencies for the first twelve modes have been given in Table 3. The first two deformed modal shapes are lateral, having exactly the same periods of 0.101 s, while the third one is torsional with a period of 0.071 sec, slightly smaller than the first two modes.  Figure 7 shows the first and third mode shapes of the tomb.

Zülfikar et al. reported that the predominant frequency of the local site, Baglarbası, Istanbul (Station R13), where the Tomb resides closely is greater than 5 Hz based on the acceleration data analyses of recent earthquakes [31]. Since the ground around the cemetery is relatively strong, vibration frequencies of the primary modes of the tomb are close to the predominant frequency of the ground. This might cause resonance and thus increase the structure's vibrational response. In other words, the first two vibration frequencies of 9.9 Hz of the monument could be well close to the predominant vibration frequency of the surrounding ground (>5 Hz).

### 6.3. Linear Time History Analysis

**Figure 8:**
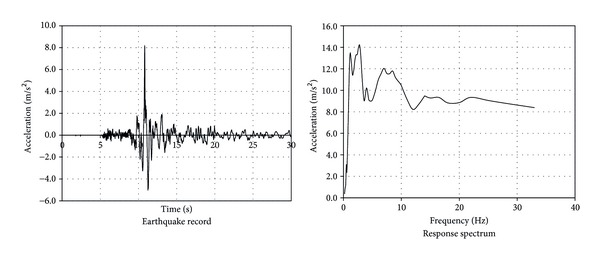
Record and response spectrum of Duzce, Turkey earthquake (November 12, 2012).

The effect of earthquake loading is critical. Therefore a step-by-step analysis to determine the dynamic response of the tomb to the Duzce Earthquake loading (Table 1) was conducted.  Figure 8 shows the earthquake record and response spectrum. It is seen that the peak ground acceleration of 8.04 m/s^2^ is quite high while the effect of the relatively high acceleration portion on the structure is relatively short. On the other hand, the response spectrum of the earthquake loading demonstrates that the primary potential resonating frequencies of the ground motion are 2.8 Hz and 8.5 Hz, which is congruent with the ground vibrations of the local site [31]. The frequency of 8.5 Hz is critically close to the tomb's first two vibration frequencies.

**Figure 9:**
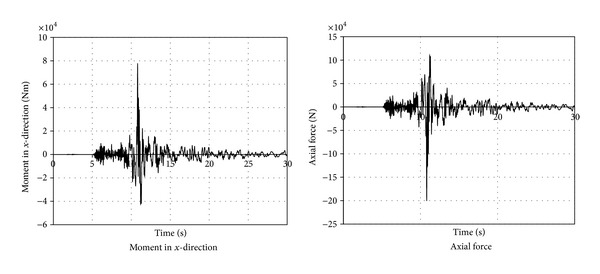
Moment and axial force time histories of the column.

While the internal compressive axial force due to the gravitational loads is 61675 N at the top of the column and 80730 N at the bottom of the column, the earthquake loading causes the column to experience internal compressive axial force of 200114 N.  Table 4 shows the maximum (envelope) internal force values developed in a column in the *x*-direction and Figure 9 shows their time histories. The figure provides the opportunity to see the variation of internal forces with time during the earthquake event.

For the columns, considering the bending moment and the axial force due to earthquake and gravitational loads, (6) helps calculate the maximum vertical tensile stresses as 4.73 MPa at the top of the column and 11.51 MPa at the bottom of the column:

(6) $$\begin{array}{c} \sigma_{z}=\frac{N}{A}+\frac{M}{I}y, \end{array}$$

where *N* is the axial force, *M* is the bending moment, *A* is the cross-sectional area, and *I* is the moment of inertia of the cross-section of the column. Developed tensile stresses are around the upper limit of the tension strength of the masonry materials. This implies that the tomb materials at roof-column junction may not be sufficiently strong and may well be in need of strengthening. Similarly, Turk [18] also used the earthquake ground motion record of Duzce Earthquake and obtained the critical highest tensile stresses as 23.4 MPa at the transition zones between the lower part and footing of the minaret, which is almost twice the highest tensile stress obtained in this study due primarily to the fact that the minaret is much taller than the tomb.

It would be noteworthy that the earthquake loads are critical for the structure taking into consideration the geometry and gravitational load distribution on it. The relatively massive and heavy roof on top of six columns creates large internal bending moments in the columns. Brittle masonry materials with an inherently low capacity to accommodate tensile stresses are vulnerable to lateral earthquake loads. This is also confirmed by observing the lateral displacements of the structure. For example, at the apex of the structure, while the lateral and vertical displacements of 2.97 mm and ~0 mm due to earthquake are obtained, only ~0 mm and 0.06 mm displacements, respectively, occur due to gravitational loads. This also accentuates the vertical rigidity and lateral flexibility of the tomb.

### 6.4. Validation of the Finite Element Results

**Figure 10:**
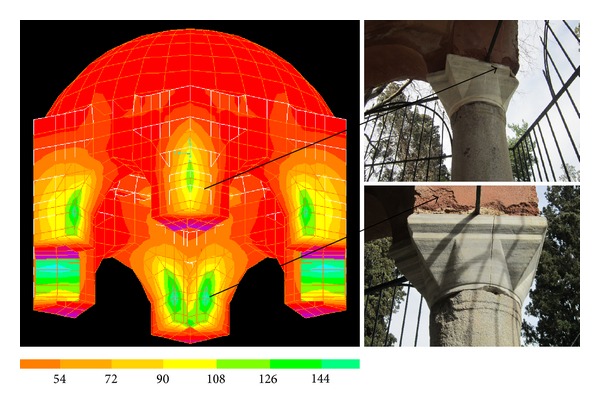
Maximum stresses in the roof (MPa) and related damage.

Comparing the computed stress distributions to the observed fractures on the structure, the FEM results were validated to some extent. The tensile stress values herein are critical especially for the brickwork as the arches connected to the granite column tops are made of brickwork. High maximum stresses show that the safety factor is unfortunately not sufficient.  Figure 10 notably supports this. For example, maximum stresses from the time history analysis at a time suggests that the damage in the upper column ends and lost brickwork seems to be the result of tensile stresses from earthquake loading. Repetitive action of this kind may cause further deterioration. Cracks in the caps could also have been originated from high bending moments and compressive forces as the masonry cannot accommodate shear stresses. The impact of the environmental weathering effects can further be observed on the structure such as surface erosion, cracks, and even fabric loss (Figures 2 and 10). This makes the situation even more critical, concerning the future state of the tomb.

## 7. Conclusions 

Many monumental tombs in various cemeteries are regarded as vital components of the cultural heritage of a society since they contain artistic, spiritual, commemorative, and social elements. This imbues them with some unique magnitude of significance and thus value, the determination of which is paramount in risk assessment. One such cultural monument, the Nişancı Hamza Paşa tomb, has been standing in Karacaahmet Cemetery, Istanbul, for centuries. As a result of this earthquake-prone locality and varying atmospheric conditions, the tomb has been exposed to various earthquakes and environmental deterioration. This might pose a threat to the future state of the tomb. Therefore, this paper presents an evaluation of the value of the tomb with respect to the historical, communal, evidential, and aesthetic aspects. The tomb has a great historical and evidential value as it is the grave of an Ottoman marksman Nişancı Hamza Paşa and was constructed in 1605. Every day, many people visit this holy place and pray to God and believe that their wish will be granted, which gives this monumental structure a considerable communal value. The aesthetic value of the tomb is notable since its brick arches, the masonry tambour made of limestone and the granite columns match very well and have an elegant appearance. Finite element simulation of the tomb was generated to assess its vulnerability. Static stress, modal, and time history analyses of the tomb employing the Duzce Earthquake (1999) record with the measured material properties of the tomb through Schmidt hammer testing show that minimum or maximum stresses developed in the dome are not too large. However, minimum stresses at locations where the arches meet the columns and maximum stresses at the top and bottom of the columns need attention. Stresses as a result of earthquake loading make the slender columns vulnerable. Developed tensile stresses are around the upper limit of the tension strength of the masonry materials, which implies that the tomb materials at roof-column junction may not be sufficiently strong and strengthening may well be required. Damage in the upper column ends, cracks in the caps, and lost brickwork seem to be in agreement with the analysis results. Therefore, further tests and analyses are needed to strengthen the structure and to prevent potential damage to the tomb.

## Figures and Tables

**Table 1:** Characteristics of the earthquake used in the analysis.

Earthquake	Record/component	PGA (g)	PGV (cm/s)	PGD (cm)	Mw	Joyner-Boore dist. (km)	Closest dist. (km)	Site class (NEHRP)	Faulting
Duzce, Turkey, November 12, 1999	DUZCE/BOL090	0.82	62.10	13.55	7.14	12.02	17.6	D	0

**Table 2:** Physical properties of tomb materials.

	*E*, modulus of elasticity	*ν*, poisson ratio	*γ*, unit weight	*f*_*c*_, compressive strength	*f*_*t*_, tensile strength	*G*, shear modulus
Granite	54 GPa	0.25	26 kN/m^3^	183 MPa	2.5–5 MPa	21.6 GPa
Marble	26 GPa	0.25	23 kN/m^3^	71 MPa	0.5–4 MPa	10.4 GPa
Stone masonry	13 GPa	0.25	20 kN/m^3^	36 MPa	0.1–4 MPa	5.2 GPa
Brick masonry	8 GPa	0.25	18 kN/m^3^	10 MPa	0.1–3 MPa	3.2 GPa
Steel	200 GPa	0.30	78 kN/m^3^	250 MPa	400 MPa	76.9 GPa

**Table 3:** First twelve vibration periods and frequencies of the tomb.

Mode	Period (sec)	Frequency (cyc./sec)	Frequency (rad./sec)
1	0.101469	9.855195	61.92201642
2	0.101468	9.855278	61.92253793
3	0.071282	14.028751	88.14524216
4	0.01965	50.891389	319.7600276
5	0.01965	50.891486	319.7606371
6	0.01626	61.500071	386.4163425
7	0.016117	62.047988	389.8590065
8	0.014341	69.731900	438.1384495
9	0.01434	69.733016	438.1454616
10	0.012712	78.666875	494.2785532
11	0.012711	78.670355	494.3004186
12	0.011164	89.575670	562.8205336

**Table 4:** Maximum (envelope) internal forces of a column due to earthquake loading.

	Moment in *x*-direction (Nm)	Axial force in (N)	Shear in *x*-direction (N)
At the top	77480.1	200114	81520
At the bottom	183385.2	200114	81520
